# Pain Assessment and Need for Analgesics after Scaling and Root Planing in Patients with Stage II and Stage III Periodontitis

**DOI:** 10.3390/medicina59071203

**Published:** 2023-06-26

**Authors:** Khalid Gufran, Mohammad Shoyab Khan, Abdullah Saad Alqahtani, Banna Alnufaiy

**Affiliations:** Department of Preventive Dental Sciences, College of Dentistry, Prince Sattam Bin Abdulaziz University, Alkharj 11942, Saudi Arabia; ms.khan@psau.edu.sa (M.S.K.); ab.alkahtani@psau.edu.sa (A.S.A.); b.alnoufaiy@psau.edu.sa (B.A.)

**Keywords:** analgesic consumption, dentistry, MDAS, periodontology, SRP, stages of periodontitis

## Abstract

*Background and Objectives*: The most common treatment procedure for periodontitis and gingivitis is scaling and root planing, which is perceived as a painful dental treatment. The current study aimed to assess pain perception and analgesics consumption after scaling and root planing (SRP) in patients with stage II and stage III periodontitis. *Materials and Methods:* Before starting the SRP, all the periodontal parameters, such as probing depth (PD), bleeding on probing (BOP), and clinical attachment level (CAL), were measured. The anxiety level of the patients was also assessed using the modified dental anxiety scale (MDAS) questionnaire. Pain perception of the patients was recorded with the visual analog scale (VAS) after performing the SRP. Patients were asked to mark their pain level on the VAS sheet after two hours, four hours, eight hours, 24 h, and 48 h after the periodontal treatment. The following cut-off points were used for the pain intensity in the VAS: 0 = no pain, 1–4 = mild pain, 4–6 = moderate pain, and 7–10 = severe pain. Patients were advised to take analgesics if the pain was intolerable. Multivariate logistic regression was performed to conduct the association of all dependent variables and the pain perception of patients. A nonparametric Friedman test was conducted to assess pain perception at different times. *Results:* A total of 52 patients including 32 males and 20 females participated in the current study, with a mean age of 43.10 ± 12.33 years. Multivariate analyses showed that MDAS and analgesic consumption is significantly associated with pain perception. Other clinical variables are not associated with pain perception. The Friedman test exhibited that pain perception is significantly associated (*p* < 0.05) with time. *Conclusions:* Analgesic consumption and anxiety level are significantly associated with pain perception after SRP treatment.

## 1. Introduction

Pain is defined as an emotional experience along with unpleasant senses which are associated with the potential damage of the tissue [1]. Most periodontal diseases are instigated by chronic inflammation which also triggers periodontium damage. Different non-surgical and surgical procedures are performed in periodontitis [2]. Fear of pain during dental treatment is commonly observed among the majority of patients. The intensity of pain and different variables that influence pain should be comprehended, as stress levels and compliance of the patients during the dental treatment could be affected by the perception of pain [3]. Therefore, clinicians should explain to the patients the level of pain during or after any dental procedure to aid patients build their expected level of pain [4].

Pain is a complicated sensory process. Previous studies stated that many factors such as previous experience and anxiety influence pain perception [5,6]. However, different dental treatments cause different pain perceptions and anxiety. The most common treatment procedure for periodontitis and gingivitis is scaling and root planing (SRP) which is perceived as a painful dental treatment [7]. The clinicians should perceive the intensity of pain experienced by the patients [8]. One of the most reliable and valid pain assessment tools is visual analog scales (VAS), which have been used in previous studies to assess the pain perception of patients in different types of dental treatments [9,10,11]. Moreover, pain perception was also assessed in different surgical and non-surgical periodontal procedures, such as gingivectomy, SRP, implant surgery, and open flap with osseous resection, to name but a few [4,7,8,11]. However, the perception of pain also depends on other associated factors, such as the consumption of analgesics. In current times, analgesics are easily available over the counter and people used to take analgesic medications for the slightest pain [12]. Therefore, it is imperative to assess the analgesic consumption along with the pain perception.

In addition, the treatment of any periodontal disease is based on the classification of the periodontitis. The classification of periodontal diseases and conditions was published in 1999 [13]. However, the previous classification was modified and changes were made in 2017 [14]. As per the literature, no previous study had been carried out to assess analgesic consumption along with the frequency and intensity of pain after nonsurgical periodontal therapy. Moreover, associating these factors with the extent and severity of the new classification of periodontitis is important. Therefore, the current study aimed to assess pain perception and analgesics consumption after SRP in patients with stage II and stage III periodontitis.

## 2. Materials and Methods

The current prospective study was conducted in the College of Dentistry, Prince Sattam Bin Abdulaziz University. The Standing Committee of Bioethics Research (SCBR) of Prince Sattam Bin Abdulaziz University approved this study protocol (SCBR-092-2022). Moreover, the study was conducted according to the guidelines of the Declaration of Helsinki. 

All the patients included in this study followed the convenience sampling method. The following inclusion criteria were used to include all the patients in the current study: patients, aged 20 to 65 years, who were diagnosed with the Class II and Class III stages of periodontitis in at least 12 teeth, as well as patients who required oral prophylaxis and non-surgical therapy. However, patients who did not receive any prior periodontal surgical therapy were included in the current study. On the other hand, pregnant and lactating patients, patients who had taken anti-depressants or sedatives, analgesics 24 h before the treatment, mentally challenged patients, patients who had periodontal therapy with antibiotics within the last three months, smoker patients, and patients who testified acute and chronic pain before starting the treatment were excluded from the current study.

The study was explained to all the patients who fulfilled the inclusion and exclusion criteria and informed consent was obtained from all the participants. The demographic data of the patients were recorded before starting the periodontal treatment. The anxiety levels of the patients before starting the treatment were assessed using the modified dental anxiety score (MDAS) questionnaire [15].

Routine periodontal clinical examination was performed by evaluating probing depth (PD) [16], bleeding on probing (BOP) [17], and clinical attachment level (CAL) [18]. All the clinical parameters were recorded only once regardless of the number of scaling sessions. PD was measured with UNC 15 (Hu-Friedy, Chicago, USA) probe by inserting the probe parallel to the long axis of the tooth in order to grasp the deepest point of the pocket. The distance between the gingival margin and the base of the pocket was recorded. The greatest probing depth was recorded for each patient. A UNC 15 (Hu-Friedy, Chicago, IL, USA) probe was also used to measure the CAL from the distance between the cementoenamel junction and the apical end of the probe. The greatest CAL was recorded for each participant. The BOP was recorded by using a gentle insertion of the periodontal probe tip into the sulcus, followed by a gentle sweep around from the proximal surface to the proximal surface. Any bleeding seen 30 s after removing the probe tip was recorded. BOP based on the percentage of sites for all teeth of a subject other than teeth without clinical crowns was measured. All the examination was carried out by a specialist periodontist. After recording the periodontal data, subgingival scaling was performed.

A visual analog scale (VAS) was used to measure the pain after the periodontal treatment. This scale consists of a horizontal line starting from zero to 10 where zero indicates no pain and 10 indicates the intense level of pain. Patients were asked to mark their pain level on the VAS sheet after 2 h, 4 h, 8 h, 24 h, and 48 h after the periodontal treatment. The following cut-off points were used for the pain intensity in the VAS: 0 = no pain, 1–4 = mild pain, 4–6 = moderate pain, and 7–10 = severe pain [19]. Patients were advised to take analgesics if the pain was not tolerable. The records of analgesic consumption were taken after a week of the treatment. 

### Statistical Analysis

All the statistical analyses were performed using the SPSS software, version 27 (IBM, Armonk, NY, USA). The normality of the data was checked using the Shapiro–Wilk test. Descriptive data were analyzed with frequency distribution. Multivariate logistic regression was performed to conduct the association of all dependent variables and the pain perception of patients. A chi-square test was performed to identify the distribution of MDAS and analgesic consumption with gender. A nonparametric Friedman test was conducted to assess pain perception at different times. When the Friedman test was significant, pairwise comparisons based on the Wilcoxon rank test were made, implemented with Bonferroni correction. Statistical significance was set at 0.05.

## 3. Results

**Figure 1:**
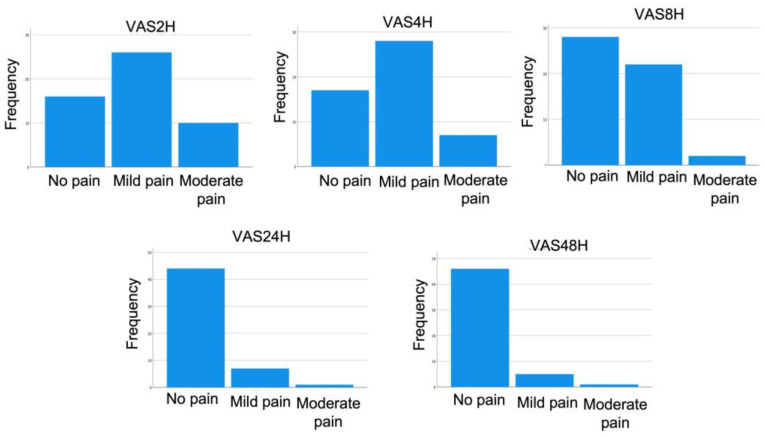
Frequency of pain at different time points.

A total of 52 patients including 32 males and 20 females participated in the current study with a mean age of 43.10 ± 12.33 years. A total of 33 and 19 patients were diagnosed with stage II periodontitis (63.50%) and stage III periodontitis (36.60%), respectively. Moreover, a total of 19, 21, and 12 patients were diagnosed with grade A, grade B, and grade C periodontitis, respectively. The majority of the patients were not anxious (65.4%) before the treatment and 80.80% of patients did not take analgesics after the SRP treatment. None of the patients experienced severe pain after the treatment. Mild and moderate pain were observed throughout 48 h. However, the majority of the patients recorded ‘no pain’ after the treatment. The frequency of the pain was displayed in Figure 1. All the descriptive data of all participants was presented in Table 1.

**Figure 2:**
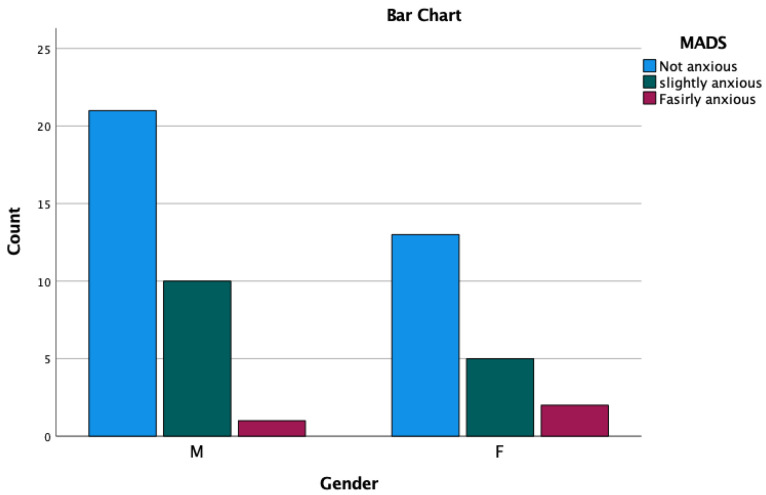
MDAS distribution between gender.

**Figure 3:**
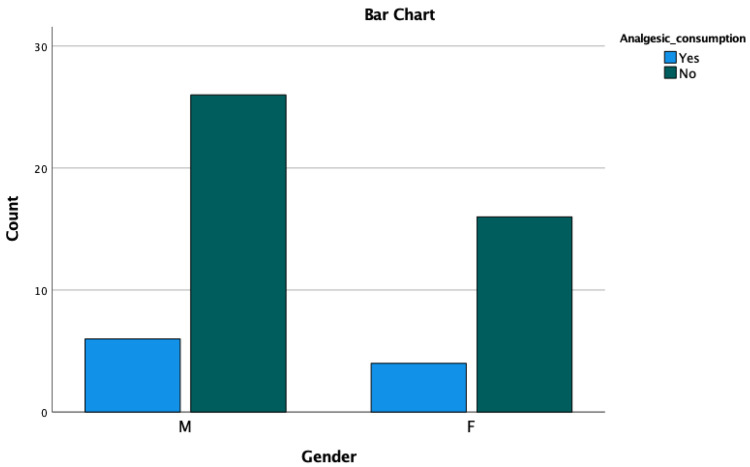
Analgesic consumption between gender.

Multivariate analyses showed that MDAS and analgesic consumption is significantly associated with pain perception. Other clinical variables are not associated with pain perception (Table 2). Moreover, the chi-square test revealed that male patients are less anxious than female patients; however, no significant differences were observed (Table 3 and Figure 2). In terms of analgesic consumption, a total of 19.20% of participants took the analgesics after treatment and the majority of the patients did not take any analgesics. No significant difference was observed between gender related to analgesic consumption (Table 4 and Figure 3).

The Friedman test exhibited that pain perception is significantly associated (*p* < 0.05) with time (Table 5). Therefore, a pairwise comparison was conducted and found that pain perception was significantly different (*p* < 0.05) with each time difference, except for 24 H and 48 H (Table 6).

## 4. Discussion

This study aimed to assess pain perception and analgesics consumption after SRP in patients with stage II and stage III periodontitis. The outcome of the study showed that analgesic consumption and dental anxiety are associated with pain. Moreover, pain is also associated with time, except for 24 H–48 H. 

The current study showed that the pain perception of males and females is not significantly different at the time point. Even though it was mentioned in the previous study that the pain scale for female patients was higher than that of male patients [20], the current study opposes that statement. The outcome of this study related to pain perception between gender is also supported by the previous studies [15,21]. The age of the patients Is also related to the perception of pain. It stated that older patients are less prone to experience pain compared to younger people, as the nociceptors are gradually lost with age [11]. However, a contrasting outcome was also observed, where the VAS score was less in younger people compared to the elderly [15]. This contrasting outcome might be due to another underlying condition which was not assessed during the study. The current study did not focus on the different age groups, though patients with a wide range of ages (20 to 65 years old) were included in this study. It showed that age is not significantly associated with pain perception in the current population. 

As stated in the previous studies, female patients are more anxious about dental treatment compared to male patients [22,23,24]. However, the contrasting report was also found where females showed insignificant lower MDAS scores compared to males [15], which might be due to the sample size and the age range of female patients who participated in the study. In this study, it exhibited that female patients were more anxious, but the difference in anxiety levels between males and females was insignificant. However, the total sample between males and females is different. Male participants are more than female participants (male = 32 and female = 20), which might result in more MDAS scores in male patients compared to female patients. A similar result was observed in the study by Singh et al. [15]. However, unlike this study, the mean age of the female participants is higher compared to the male participants in the study by Singh et al. [15]. Due to the higher mean age of the female patients, female patients showed less anxiety than their male counterparts in a few different studies [15,25,26]. Even though the MDAS score did not show any significant difference between gender, the current study showed that MDAS is significantly associated with VAS at different time points. Therefore, it could be indicated that dental anxiety is linked to the perception of pain.

The perception of pain is usually linked to the severity of the periodontal disease. The more aggressive the periodontal condition, the more pain experienced by the patients [27]. The current study focused on stage II and stage III of periodontitis, where no advanced treatment is required other than SRP. The outcome of the study exhibited that pain scores using VAS did not show significant differences with the stages and grades of periodontitis. A similar outcome was also observed in the previous studies [19,28]. However, some studies on periodontal pain did not assess pain perception with periodontal conditions [29,30].

Periodontal treatment procedures might have associated with the level of pain which results in the consumption of analgesics oftentimes. In this study, analgesics were prescribed to patients if the pain was intolerable. It showed that 20% and 18.80% of female and male patients were taking analgesics after the SRP treatment. However, the majority of the patients did not take medication for pain. Even though more female patients were taking analgesics compared to male patients, the ratio was not significant, and no significant differences were observed between analgesic consumption and gender. Experienced practitioners play an imperative role in postoperative pain. SRP could be painful if patients are not treated sophistically which could lead to analgesic consumption. In the current study, all the professional periodontists performed the SRP procedure which might report a lower percentage of analgesic consumption. Although analgesic consumption is not related to gender, the statistical analysis of this study exhibits that analgesic consumption is significantly associated with VAS at different time points. 

The current study used the VAS for assessing the pain perception of patients after the SRP treatment which is one of the most common tools measuring pain used in many previous studies not only in periodontal treatment but also in other branches of dentistry [2,15,19,28,31]. Another pain-assessing tool numeric rating scale (NRS) also been used in different studies [4,9,32,33,34]. However, the NRS pain assessment tool usually using for younger patients due to easy understanding. In this study all the patients were adults; hence, no precautions in choosing the scaling method were taken, and followed the extensively accepted VAS method.

The patients used the VAS questionnaire to mark their pain level at 2, 4, 8, 12, and 48 h after the treatment. It is obvious that pain would be intense at the nearest hours after the treatment and would gradually decrease. The outcome of the study also showed the same, where after two hours the mean pain score was 0.88 and decreased to 0.13 after 48 h. A similar report was also observed in the previous studies irrespective of the time point of assessing the pain [2,19,31]. This study found a significant decrease in pain perception over time. Moreover, a significant decrease was observed from each time period such as two hours to four hours, four hours to eight hours, and eight hours to 24 h. No significant difference was observed between 24 h to 48 h. After 24 h of the treatment, no pain was observed by the majority of the patients. 

The current study measured the BOP, CAL, and PD as a routine radiographic examination of the periodontal treatment. CAL and PD are used to identify the classification of periodontal stages and grades. Moreover, BOP is a good indicator of active periodontal disease [14]. This study assessed the periodontal variables with pain perception at different times. However, no significant association was observed between periodontal variables and pain perception. This also justifies the insignificant outcome for the stages and grades of periodontitis, where the VAS scores such as CAL, PD, and BOP are directly linked with the stages and grades of periodontitis. A similar outcome was also found in the previous study by Palheiros et al. [19] who assessed the periodontal variables with pain perception. However, the periodontal parameter usually only assesses for the routine examination for the periodontal treatment. The optimum pain perception of patients could be attained after active periodontal treatment. Additionally, other therapies such as ozone [35], laser photodynamic therapy [36], and regenerative materials [37] could have a significant influence on the oral environment. These therapies could modify clinical and microbiological parameters in periodontal patients and could have an effect on pain assessment in combination with SRP therapy. All these variables should be considered in future trials.

The limitation of the current study regarding the sample size calculation is that all the patients were included as a convenient sampling method. However, a proper sample size calculation would have provided a precise outcome. Moreover, the age range of the included patients was extensive. A specific age range could provide an improved outcome for this study. Therefore, further studies with enlarged samples and specific age ranges should be conducted.

## 5. Conclusions

Analgesic consumption and anxiety levels are significantly associated with pain perception after SRP treatment.

## Figures and Tables

**Table 1:** Descriptive data of the participants.

Variables	N	Percent (%)
Gender		
Male	32	61.50
Female	20	38.50
Age (Mean ± SD)	43.10 ± 12.33 years	
BOP (Median ± IQR)	44.00 ± 20.00%	
PD (Median ± IQR)	5.00 ± 2.00 mm	
CAL	3.00 ± 2.00 mm	
Periodontitis		
Stage II	33	63.50
Stage III	19	36.50
Grade		
Grade A	19	36.50
Grade B	21	40.40
Grade C	12	23.10
MDAS		
Not Anxious	34	65.40
Slightly anxious	15	28.80
Fairly anxious	3	5.80
Analgesic consumption		
Yes	10	19.20
No	42	80.80
VAS		
2 H		
No pain	16	30.80
Mild pain	26	50.00
Moderate pain	10	19.20
Severe pain	0	0
4 H		
No pain	17	32.70
Mild pain	28	53.80
Moderate pain	7	13.50
Severe pain	0	0
8 H		
No pain	28	53.80
Mild pain	22	42.30
Moderate pain	1	1.90
Severe pain	0	0
24 H		
No pain	44	84.60
Mild pain	7	13.50
Moderate pain	1	1.90
Severe pain	0	0
48 H		
No pain	46	88.50
Mild pain	5	9.60
Moderate pain	1	1.90
Severe pain	0	0

N, total number; SD, standard deviation; IQR, interquartile range; MDAS, modified dental anxiety stress; BOP, bleeding on probing; PD, pocket depth; CAL, clinical attachment level; VAS, visual analog scale; H, hours.

**Table 2:** Multivariate analysis of variables and pain perception.

Pain	Variables	F	df	P
VAS 4 H	Gender	0.42	2	0.660
	Age	0.85	2	0.435
	Periodontal stage	0.50	2	0.608
	Periodontal grade	1.49	2	0.236
	BOP	1.01	2	0.372
	PD	0.39	2	0.675
	CAL	0.30	2	0.741
	MDAS	7.02	2	0.002 *
	Analgesic consumption	49.38	2	0.0001 *
VAS 8 H	Gender	2.41	2	0.100
	Age	1.79	2	0.176
	Periodontal stage	1.05	2	0.357
	Periodontal grade	0.35	2	0.704
	BOP	1.19	2	0.313
	PD	0.24	2	0.790
	CAL	0.70	2	0.500
	MDAS	5.35	2	0.008 *
	Analgesic consumption	5.75	2	0.006 *
VAS 24 H	Gender	2.15	2	0.127
	Age	0.73	2	0.489
	Periodontal stage	1.20	2	0.309
	Periodontal grade	0.59	2	0.558
	BOP	0.51	2	0.606
	PD	0.41	2	0.664
	CAL	1.22	2	0.304
	MDAS	4.69	2	0.014 *
	Analgesic consumption	7.69	2	0.001 *
VAS 48 H	Gender	2.34	2	0.107
	Age	1.03	2	0.366
	Periodontal stage	1.99	2	0.148
	Periodontal grade	1.02	2	0.367
	BOP	0.23	2	0.794
	PD	0.09	2	0.907
	CAL	1.57	2	0.219
	MDAS	5.93	2	0.005 *
	Analgesic consumption	3.23	2	0.048 *

F, F statistics; df, degree of freedom; P, *p*-value; VAS, visual analog scale; H, hours; BOP, bleeding on probing; PD, pocket depth; CAL, clinical attachment level; MDAS, modified dental anxiety stress; *, statistically significant (*p* < 0.05).

**Table 3:** Frequency of the MDAS between gender.

Gender	MDAS (%)			P
	Not Anxious	Slightly Anxious	Fairly Anxious	
Male	21 (65.60)	10 (66.70)	1 (3.10)	0.556
Female	13 (65.00)	5 (25.00)	2 (10.00)	

MDAS; modified dental anxiety score, %; percentage, P; *p*-value.

**Table 4:** Frequency of analgesic consumption between gender.

Gender	Analgesic Consumption (%)		P
	Yes	No	
Male	6 (18.80)	26 (81.30)	1.000
Female	4 (20.00)	16 (80.00)	

%; percentage, P; *p*-value.

**Table 5:** Comparison of pain perception over time.

VAS	Mean	SD	df	P
2 H	0.88	0.70	4	0.0001 *
4 H	0.81	0.66		
8 H	0.50	0.58		
24 H	0.17	0.43		
48 H	0.13	0.39		

VAS, visual analog scale; H, hours; SD, standard deviation; df, degree of freedom; P, *p* value; *, a significant difference (<0.05).

**Table 6:** Pair-wise comparison of different times.

VAS Time	Z	P
2 H–4 H	−2.000	0.046 *
2 H–8 H	−3.879	0.0001 *
2 H–24 H	−5.476	0.0001 *
4 H–8 H	−3.557	0.0001 *
4 H–24 H	−5.296	0.0001 *
4 H–48 H	−4.359	0.0001 *
8 H–24 H	−4.123	0.0001 *
8 H–48 H	−4.359	0.0001 *
24 H–48 H	−1.414	0.157

VAS, visual analog scale; H, hours; Z, z statistics; P, *p* value; *, significant difference (<0.05).

## Data Availability

The data presented in this study are contained within the article.
